# The 1‑year safety and efficacy outcomes of Absorb bioresorbable vascular scaffolds for coronary artery disease treatment in diabetes mellitus patients: the ABSORB DM Benelux study

**DOI:** 10.1007/s12471-019-1293-7

**Published:** 2019-06-13

**Authors:** T. M. Hommels, R. S. Hermanides, S. Rasoul, B. Berta, A. J. J. IJsselmuiden, G. A. J. Jessurun, E. Benit, B. Pereira, G. De Luca, E. Kedhi

**Affiliations:** 1 Isala Klinieken, Zwolle, The Netherlands; 2 Zuyderland Medisch Centrum, Heerlen, The Netherlands; 3 Albert Schweitzer Ziekenhuis, Dordrecht, The Netherlands; 4 Scheper Ziekenhuis, Emmen, The Netherlands; 5 Virga Jesse Ziekenhuis, Hasselt, Belgium; 6 Institut National de Chirurgie Cardiaque et de Cardiologie Interventionnelle, Luxembourg, Luxembourg

**Keywords:** Bioresorbable scaffold, Diabetes mellitus, Coronary artery disease, Percutaneous coronary intervention, Scaffold thrombosis

## Abstract

**Background:**

Diabetes mellitus (DM) patients show higher rates of repeat revascularisation even in the era of modern drug-eluting stents (DES). The concept of bioresorbable scaffolds is becoming captivating, as it might allow for repeat interventions, prolonging the time span during which patients can be treated by percutaneous coronary intervention (PCI).

**Aims:**

We intend to evaluate the short- and long-term safety and efficacy of Absorb bioresorbable vascular scaffolds (Absorb BVS) in the treatment of coronary artery disease (CAD) in DM patients for any indication.

**Methods:**

The ABSORB DM Benelux is an international prospective study in DM patients who have undergone PCI with ≥1 Absorb BVS. Major adverse cardiac events (MACE) at 1 year was the primary endpoint, defined as a composite of all-cause death, any myocardial infarction (MI) and ischaemia-driven target vessel revascularisation (TVR). Secondary endpoints were target lesion failure (TLF) and definite or probable scaffold thrombosis (ScT).

**Results:**

Between April 2015 and March 2017, 150 DM patients and 188 non-complex lesions were treated. Device implantation was successful in 100%. MACE occurred in 14 (9.5%) patients, with all-cause death occurring in 4 (2.7%), any MI in 6 (4.1%) and ischaemia-driven TVR in 7 (4.8%) respectively. TLF was reported in 11 (7.5%). Definite and probable ScT was observed in 2 (1.4%).

**Conclusion:**

Absorb BVS for treatment of anatomically low-risk patients with DM show acceptable safety and efficacy outcomes at 1 year. If these promising results are confirmed after a longer follow-up period, new-generation bioresorbable scaffolds combined with refinement of implantation techniques might open new horizons for CAD treatment in DM patients.

**Electronic supplementary material:**

The online version of this article (10.1007/s12471-019-1293-7) contains supplementary material, which is available to authorised users.

## What’s new?

- This is the first prospective study utilising Absorb bioresorbable vascular scaffolds for treatment of anatomically low-risk patients with diabetes mellitus for any indication.
- Acceptable safety and efficacy outcomes were obtained at 1‑year follow-up.
- A low incidence of scaffold thrombosis was observed with no occurrences of late thrombosis.
- If these promising results are confirmed after a longer follow-up period, more performant bioresorbable scaffolds might open new horizons for treatment of coronary artery disease in diabetes mellitus patients.

## Introduction

The incidence and prevalence of diabetes mellitus (DM) is increasing in both developed and developing countries [1, 2]. People with DM are between 2 and 4 times more likely to develop coronary artery disease (CAD) compared to non-DM patients [3–6]. Despite the major advances in percutaneous coronary intervention (PCI) with newer generations of drug-eluting stents (DES) accompanied by improved medical treatment, studies have continued to show a trend towards higher rates of major adverse cardiovascular events in DM patients compared to non-DM patients [7, 8].

Bioresorbable polymer drug-eluting scaffold systems enable the application of a short-term vessel scaffold (which subsequently dissolves) combined with drug delivery capability. The short-term results from the Absorb bioresorbable vascular scaffolds (Absorb BVS) clinical trial programs, at the time the study was designed, showed promising safety and efficacy outcomes for these devices, being non-inferior to those of the best-in-class durable polymer everolimus-eluting stents (EES) [9–15]. Considering the scaffold resorption, it was conceivable that vessel restoration following Absorb BVS implantation might be associated with more favourable long-term outcomes compared to metallic DES, mainly because inflammation induced by foreign bodies is only transient following Absorb BVS implantation while it is permanent after metallic DES implantation. Particularly in DM patients, where diabetes-related chronic peristrut inflammation triggers more aggressive restenosis, reduction of permanent inflammatory triggers may further improve clinical outcomes. Furthermore, repeat interventions at target lesions could be effectuated multiple times without a critical loss of vessel diameter, thus prolonging the time interval during which CAD in DM patients could still be managed by PCI. Henceforth, we designed the ABSORB DM Benelux Study to evaluate the short- and long-term safety and efficacy of the Absorb BVS in patients with DM.

## Methods

The ABSORB DM Benelux Study is an international study in patients with DM and de novo lesions treated with the ABSORB family and conducted in The Netherlands, Belgium and Luxembourg. This clinical investigation is a prospective register and did not test any new device. The study was approved in February 2015, in accordance with the Declaration of Helsinki, by the Ethical Committees of each participating centre.

### Study population

All patients aged ≥18 years with a history of DM undergoing PCI with implantation of ≥1 Absorb BVS for any indication, in a de novo lesion located in a native non-grafted artery, could be enrolled. The exclusion criteria were determined as: pregnancy; patients unable to provide (written) informed consent; known left ejection fraction <30%; life expectancy <3 years and inability to undergo dual antiplatelet therapy (DAPT) for at least 12 months.

### Endpoints and definitions

The primary endpoint was the incidence of major adverse cardiac events (MACE) at 1 year, defined as a composite of all-cause death, any myocardial infarction (MI) and ischaemia-driven target vessel revascularisation (TVR). The secondary endpoints represented target lesion failure (TLF), defined as a composite of cardiac death (CD), target vessel MI and ischaemia-driven target lesion revascularisation (TLR), the incidence of definite or probable scaffold thrombosis (ScT) and the 1‑year incidence rates of the endpoint composites. Adverse event definitions are described in the Supplementary Table.

### PCI procedure

The implanted devices are the bioresorbable polymer drug-eluting scaffold ABSORB BVS system and the ABSORB GT1 system (Abbott Vascular, Santa Clara, CA, USA). These devices are composed of poly-L-lactic acid and an everolimus-eluting polymer coating of poly-dl-lactic-acid, both of which are completely bioresorbable through a natural metabolic process within 3 years [16]. The average strut thickness is 150 µm. The device was available in diameters ranging from 2.5 to 3.5 mm with a length of 8, 12, 18, 24, or 28 mm. Implantation of an Absorb BVS was at the discretion of the operator. The vessel size, similar to other trials with this particular device, ranged from 2.50 to 3.75 mm. Predilatation and postdilatation were strongly recommended. Intracoronary imaging by means of optical coherence tomography (OCT) or intravascular ultrasound (IVUS) was encouraged but not mandatory. Treatment of bifurcations was not encouraged; however, in this case a provisional T‑stenting technique was advised. There were no limitations regarding lesion length; however, treatment of very calcified and tortuous lesions was not encouraged. If found necessary, additional implantation of metallic DES was accepted as a bailout procedure. Angiographic success was defined as a visually assessed <30% residual stenosis of the target lesion after successful device implantation. Procedural success was defined as angiographic success with no occurrence of events during the procedure. All patients received DAPT for at least 12 months.

### Follow-up and assessment of adverse events

Clinical follow-up included clinical visits and telephone contact. All reported adverse cardiac events underwent assessment by an independent clinical event committee (Diagram BV, Zwolle, The Netherlands). Angiographic evaluations of baseline as well as repeat angiograms in patients with events were analysed by means of quantitative coronary angiography, Thrombolysis in Myocardial Infarction (TIMI) as well as presence of thrombus analysis by an independent core laboratory (Diagram BV, Zwolle, The Netherlands).

### Statistical analysis

The baseline clinical and angiographic characteristics are presented using descriptive statistics. Categorical variables are summarised as frequency and percentages. Continuous variables are summarised as mean and standard deviation. The composite endpoints and clinical events are presented by using the Kaplan-Meier survival method with time-to-event analysis. In addition, a multivariate Cox regression model with adjustment for age, gender, PCI indication (acute coronary syndrome versus non-acute coronary syndrome) and insulin-treated DM was performed and presented with hazard ratio (HR) and 95% confidence interval (CI). Other regression models were performed for relevant factors such as multivessel or multiple lesion treatment (≥2 vessels/lesions), proximal versus distal segment implantation (proximal coronary location—segment number 1, 5, 6, 11 versus none), number of devices used for target lesion, total length of implanted devices, employment of intracoronary imaging and utilisation of pre- and postdilatation. A *p*-value <0.05 was considered to indicate formal statistical significance. The analyses were conducted with intention-to-treat. All statistical analyses were performed using SPSS version 25 (IBM Corp., Armonk, NY, USA).

## Results

Between April 2015 and March 2017, a total of 150 DM patients and 188 lesions were treated by PCI with implantation of Absorb BVS. The patients were treated in 18 different centres by experienced Absorb BVS operators. Their baseline clinical characteristics are shown in Tab. 1. The patients had a mean age of 63.4 ± 10.4 years and were predominantly male (72%). DM type II was diagnosed in 93.3% and insulin-treated DM at hospital admission was 31.3%.

**Table 1:** Clinical characteristics of the patients at baseline

Baseline clinical characteristic	Patients (*n* = 150)
Age (years)—mean ± SD	64.3 ± 10.4
Sex (male)—*n* (%)	108 (72.0)
Race (Caucasian)—*n* (%)	140 (93.3)
Body-mass index (kg/m^2^)—mean ± SD; *n*	29.5 ± 5.1;*148*^a^
*Risk factors—n (%)*	
Diabetes mellitus type 1	10 (6.7)
Diabetes mellitus type 2	140 (93.3)
Insulin-dependent diabetes mellitus	47 (31.3)
Diabetes mellitus treated with oral antidiabetic	117 (78.0)
HbA1c at hospitalisation (mmol/mol)—mean ± SD; *n*	55.5 ± 11.5;*42*^a^
Arterial hypertension	104 (69.3)
Hypercholesterolaemia	100 (66.7)
Family history of cardiovascular disease	59 (39.3)
Current smoker	35 (23.3)
*Medical history—n (%)*	
Previous ACS	41 (27.3)
Previous PCI	37 (24.7)
Previous CABG	8 (5.3)
Previous CVA or TIA	10 (8.7)
Severe chronic renal failure^b^	4 (2.7)
Chronic pulmonary obstructive disease^c^	11 (7.3)
*Clinical presentation—n (%)*	
Acute coronary syndrome	73 (48.7)
ST-segment elevation myocardial infarction	18 (12.0)
Non-ST-segment elevation myocardial infarction	29 (19.3)
Unstable angina pectoris	26 (17.3)
Non-acute coronary syndrome	77 (51.3)
Stable angina pectoris	59 (39.3)
Silent ischaemia	8 (5.3)
Other	10 (6.7)

Plus—minus values are means ± standard deviation

*ACS* acute coronary syndrome, *PCI* percutaneous coronary intervention, *CABG* coronary artery bypass grafting, *CVA* cerebrovascular accident, *TIA* transient ischaemic attack

^a^Italic numbers represent the known total from which the variable was calculated

^b^Renal insufficiency was defined as estimated glomerular filtration rate of less than 30 ml/min per 1.73m^2^ of body surface area (GFR <30 ml/min/1.73m^2^)

^c^Chronic pulmonary obstructive disease was defined as ≥Gold class II

The angiographic characteristics are described in Tab. 2. The 188 target lesions were treated with a total of 214 implanted devices (ABSORB BVS 60.7%, ABSORB GT1 34.1% and metallic DES 5.1% in addition to implantation of ≥1 Absorb BVS). Device implantation was successful in 100% of the patients and the procedural success was 99.5% with a single patient developing a distal coronary dissection after implantation of an ABSORB BVS with good clinical evolution. Predilatation was performed in 93.3% and postdilatation in 75.5% of the procedures. In no case was postdilatation balloon size more than 0.5 mm larger than the scaffold size. Preimplantation, intracoronary imaging with OCT and IVUS was conducted in 7.4% and 1.0% respectively. Postimplantation, OCT was effectuated in 6.4% and IVUS in 1.6%, mostly for the purpose of apposition control. Postprocedural TIMI grade 3 was observed after all procedures.

**Table 2:** Angiographic characteristics of the patients at baseline

Baseline angiographic characteristics	
**Patient-level analysis**	
Number of patients	150
Number of treated target lesions—mean ± SD	1.3 ± 0.5
Treated target lesions ≥2—*n* (%)	30 (20.0)
Number of treated target vessels—mean ± SD	1.1 ± 0.3
Treated target vessels ≥2—*n* (%)	12 (8.0)
Devices implanted in proximal coronary segment—*n* (%)^b^	57 (38.0)
**Lesion-level analysis**	
Number of lesions	188
*Coronary artery lesion distribution—n (%)*	
Right coronary artery	57 (30.3)
Left anterior descending artery	89 (47.3)
Circumflex artery	40 (21.3)
Arterial or venous graft	2 (1.1)
*Coronary artery lesion characteristics*	
Visual estimated diameter stenosis—mean ± SD;* n*^c^	85.5 ± 11.9;*181*^a^
Bifurcation—*n* (%)	27 (14.4)
**Device-level analysis**	
Number of devices	214
*Device distribution—n (%)*	
ABSORB BVS	130 (60.7)
ABSORB GT1	73 (34.1)
Metallic DES	11 (5.1)
*Number of devices at lesion—n (%)*	
1	168 (89.4)
2	16 (8.5)
3	2 (1.1)
4	2 (1.1)
Number of devices per lesion—mean ± SD	1.1 ± 0.5
Device diameter—mean ± SD	3.0 ± 0.4
Inflation pressure—mean ± SD; *n*^d^	14.3 ± 2.6;*211*^a^
Total treated length—mean ± SD	29.7 ± 19.0
**Procedure-level analysis**	
*Results—n (%)*	
Visual diameter stenosis postprocedure <30%	*185*^a^ (100)
Postprocedural TIMI grade 3	*186*^a^ (100)
Angiographic success	188 (100)
Device implantation success	188 (100)
Procedural success	187 (99.5)
*Peri-implantation procedures*	
FFR measurement—*n* (%)	26 (13.8)
Preimplantation OCT or IVUS—*n* (%)	14 (7.4)
Predilatation—*n* (%)	177 (94.1)
Predilatation balloon size—mean ± SD; *n*	2.8 ± 0.8;*176*^a^
Predilatation pressure—mean ± SD; *n*^d^	14.8 ± 4.0;*174*^a^
Postdilatation—*n* (%)	142 (75.5)
Postdilatation balloon size—mean ± SD	3.2 ± 0.5
Postdilatation pressure—mean ± SD^d^	17.3 ± 4.3
Postdilatation balloon size >0.5 mm larger than scaffold size—*n* (%)	0
Postimplantation OCT or IVUS—*n* (%)	15 (8.0)

Plus—minus values are means ± standard deviation

Length of lesions, devices and balloons were measured in millimetres (mm), as was the diameter of the devices

*DES* drug-eluting stents, *PCI* percutaneous coronary intervention, *TIMI* Thrombolysis in Myocardial Infarction with grade 3 referenced as completely restored flow, *FFR* fractional flow reserve, *OCT* optical coherence tomography, *IVUS* intravascular ultrasound

^a^Italic numbers represent the known total from which the variable was calculated

^b^Proximal devices were defined as implantation at lesion segments 1, 5, 6, 11

^c^Visual estimated diameter stenosis was defined as a percentage

^d^Dilatation and inflation pressures were measured in atmospheres (atm)

### Clinical outcomes

**Fig. 1:**
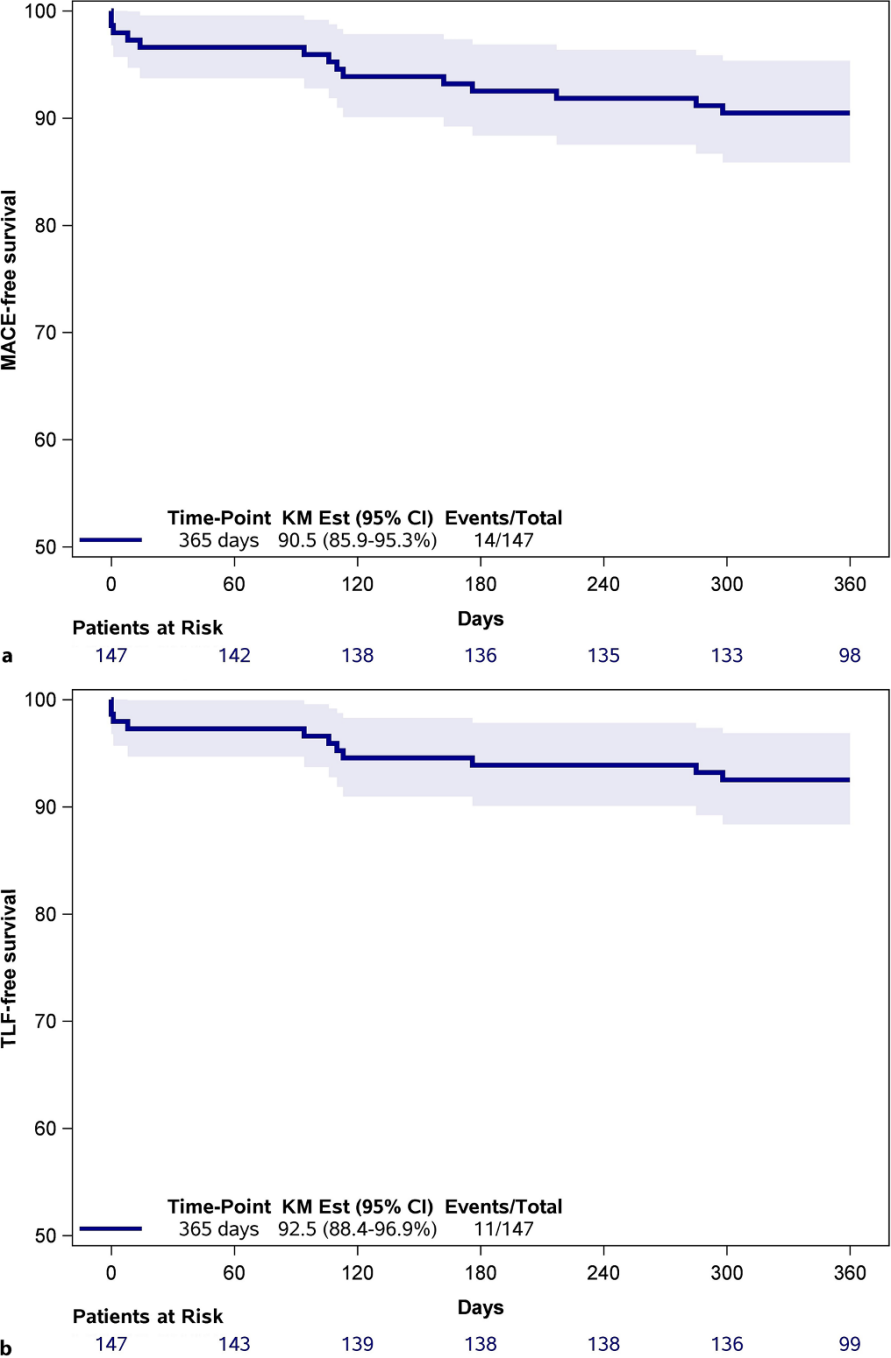
Kaplan-Meier estimates of **a** primary endpoint major adverse cardiac events and **b** target lesion failure at 1‑year follow-up. *MACE* major adverse cardiac events, *TLF* target lesion failure, *KM* *Est* Kaplan-Meier estimate, *CI* confidence interval

**Fig. 2:**
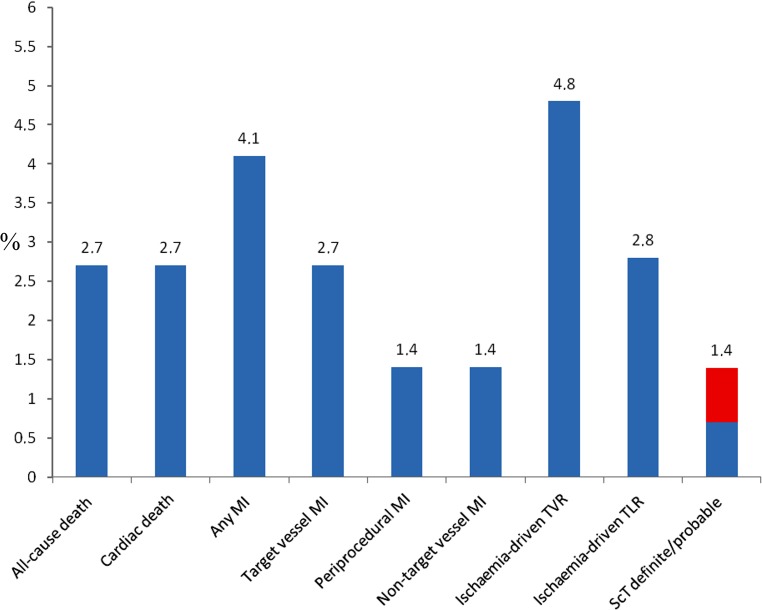
One-year Kaplan-Meier estimates of each of the composites of the endpoints. For scaffold thrombosis an explicit difference has been made between definite (*blue*) and probable (*red*), both accounting for 0.7% of the total 1.4%. There were no occurrences of late definite or probable scaffold thrombosis. *MI* myocardial infarction, *TVR* target vessel revascularisation, *TLR* target lesion revascularisation, *ScT* scaffold thrombosis

All patients received a complete 1‑year follow-up. Three patients (2.0%) were lost to follow-up during this period. At 1‑year follow-up, 72.7% of the patients were still receiving DAPT. The clinical outcomes at 1 year are presented in Tab. 3 and corresponding Figs. 1 and 2. MACE occurred in 14 patients (9.5%), with all-cause death occurring in 4 (2.7%), any MI in 6 (4.1%) and ischaemia-driven TVR in 7 (4.8%) respectively. TLF was observed in 11 patients (7.5%), with CD occurring in 4 (2.7%), target vessel MI in 4 (2.7%) and ischaemia-driven TLR in 4 (2.8%) respectively. Although 2 deaths resulted from progression of malignancy, judging them as CD could not be avoided. Of the recorded target vessel MIs, 2 (1.4%) were periprocedural. Definite and probable ScT was observed in 2 (1.4%) patients. One 80-year-old male patient had a definite ScT after treatment of a single vessel and a single lesion with an ABSORB GT1 in the proximal circumflex artery for an acute coronary syndrome. He presented with an acute MI 8 days after the procedure and underwent successful revascularisation. At the index procedure predilatation was carried out, but no postdilatation or intracoronary imaging was performed. The second patient, a 62-year-old male, with treatment of a single vessel and a single lesion with an ABSORB GT1 in the proximal left anterior descending artery for a non-acute coronary syndrome indication, died of an unknown cause the day after discharge and was regarded as a probable ScT. At the index procedure, pre- and postdilatation were conducted, but intracoronary imaging was not performed. Finally, in a multivariate Cox regression analysis adjusting for age, gender and PCI indication, insulin-treated DM was the only variable that showed a trend toward predicting MACE (HR 2.76; 95% CI: 0.94–8.07; *p* = 0.06).

**Table 3:** Safety and efficacy outcomes at 1-year follow-up

Endpoints and clinical events—% (*n*)	Patients (*n* = 147)
Primary endpoint: MACE^a^	9.5 (14)
All-cause death	2.7 (4)
Any myocardial infarction	4.1 (6)
Ischaemia-driven target vessel revascularisation	4.8 (7)
Target lesion failure^b^	7.5 (11)
Cardiac death	2.7 (4)
Target vessel myocardial infarction	2.7 (4)
Periprocedural myocardial infarction	1.4 (2)
Ischaemia-driven target lesion revascularisation	2.8 (4)
Definite or probable scaffold thrombosis	1.4 (2)
Early: 0–30 days	1.4 (2)
Acute: ≤24 h	0
Subacute: >24 h–30 days	1.4 (2)
Late: 31 days: ≤1 year	0
Very late: >1 year	0
Definite	0.7 (1)
Probable	0.7 (1)

The clinical outcomes represented as endpoints and clinical events at 1‑year follow-up. Three patients were lost to follow-up. Endpoints and clinical events are presented by Kaplan-Meier estimates. Angiographic evaluations of baseline as well as repeat angiograms in patients with events were analysed by means of quantitative coronary angiography, Thrombolysis in Myocardial Infarction as well as presence of thrombus analysis by an independent core laboratory (Diagram BV, Zwolle, The Netherlands)

^a^Major adverse cardiac events (*MACE*) were defined as a composite of all-cause death, any myocardial infarction and ischaemia-driven target vessel revascularisation

^b^Target lesion failure was defined as a composite of cardiac death, target vessel myocardial infarction and ischaemia-driven target lesion revascularisation

## Discussion

The Benelux ABSORB DM Study, the first dedicated prospective study with utilisation of Absorb BVS for percutaneous treatment of CAD in DM patients for any indication in non-complex anatomy shows acceptable safety and efficacy outcomes in this clinically high-risk patient population.

The efficacy outcomes observed in our study are similar to the 1‑year results of the ABSORB III trial with very comparable clinical outcomes for ischaemia-driven TVR and ischaemia-driven TLR [17]. Similarly, the TLF data were almost identical. Also, the reported ScT incidence was similar in both trials. Although ABSORB III included only 31.5% DM patients and no patients with ongoing MI, both studies share a relatively low lesion risk and therefore, as previously shown, simple lesions behave similarly in DM and non-DM patients after PCI [18].

Compared to 1‑year DM sub-analysis of the ABSORB trials, we observed a similar incidence of TLF, ischaemia-driven TVR and ischaemia-driven TLR. However, the incidences of the safety outcomes like ScT and target vessel MI were somewhat lower in our study [19]. While clinical and angiographic baseline characteristics were similar, the observed safety differences may be explained by the longer experience in the handling of Absorb BVS of the participating centres in our study. Indeed, postdilatation balloon size was never more than 0.5 mm larger than scaffold size, showing strict compliance with the procedure protocol.

Comparing the performance of the Absorb BVS to EES in DM patients of the SPIRIT V diabetic study, Absorb BVS has a lower incidence of MACE, TLF, ischaemia-driven TVR and TLR with comparable incidences of target vessel MI at 1‑year follow-up [20]. The patient groups had corresponding angiographic characteristics but there are non-negligible racial differences between both studies. Furthermore, our study showed similar safety and efficacy outcomes with EES used in DM patients in a pooled database of SPIRIT and COMPARE trials, as was also found in a propensity score matched comparison of the ABSORB EXTEND and the SPIRIT trials [7, 21].

The rationale for bioresorbable scaffolds derives from previous evidence that consistently reported that DM patients suffer worse outcomes with PCI as compared to coronary artery bypass grafting (CABG) [22, 23]. However, PCI is still performed in DM patients, particularly in those with single vessel disease as well as multivessel disease with a low SYNTAX score, as is consistent with the results from the SYNTAX trial [24]. Considering the accelerated nature of coronary atherosclerosis disease, CAD presents at a younger age in DM patients. Therefore, maintaining these patients free of ischaemia over decades becomes challenging. While CABG is indeed the treatment of choice for advanced multivessel disease in this patient population, it is known that venous grafts have a limited patency, giving PCI an important role in delaying the time at which these patients ultimately undergo CABG [25]. From this perspective, efforts to improve PCI outcomes in early-stage CAD in DM patients become paramount.

Furthermore, a large analysis of a pooled database of 18 clinical trials has shown that in the DES era, clinical outcomes after PCI in DM patients are highly dependent on lesion complexity at baseline with simple lesions being associated with similar efficacy outcomes to those in non-DM patients [18]. These data suggest that PCI may have favourable outcomes in a well-selected group of patients with DM, provided the extent of disease is less complex. However, progressive restenosis remains problematic with metallic DES, as the number of possible re-interventions is limited. Conversely, resorbable scaffolds are becoming attractive in this particular setting considering the theoretically larger number of possible re-interventions and thus extending the time span during which these patients could still be treated percutaneously.

On the basis of the above, the 1‑year results of our study, which showed that Absorb BVS has promising safety and efficacy outcomes post-PCI in DM patients with non-extensive CAD, stimulate further research in this direction. If maintained up to 3 years, the expected resorption period of this device, this treatment may herald brighter perspectives for PCI in DM patients. Whether this would be the case remains questionable, as the long-term follow-up safety outcomes from the AIDA and ABSORB III trials were unfavourable for Absorb BVS in comparison with EES [26, 27]. On the other hand, these trials also brought to light the importance of the scaffold implantation technique and the advantageous role of longer DAPT regimens [28, 29]. From this perspective, improved implantation techniques and newer, thinner strut resorbable devices combined with 3‑year DAPT regimens may result in improved safety outcomes. This trial was performed in The Netherlands, Belgium and Luxembourg. Following the recommendations of the Dutch Society of Cardiology, we also provided this recommendation to all participating centres in The Netherlands. All patients were recommended to continue DAPT if they were in the range of 3 years post index procedure and were not at high risk for bleeding events.

## Limitations

This study has different limitations. First, it has the general intrinsic limitations of a prospective single-arm study lacking a comparative arm. Furthermore, the study population was smaller than foreseen due to the effectuated stop in clinical utilisation of the Absorb BVS. Despite our efforts, the multivariate analysis performed was limited by the number of events observed. There was no uniform procedure protocol for scaffold implantation; however, the different participating centres had years-long experience with Absorb BVS implantation. In addition, the outcomes were not adjusted for the small number of metallic DES that were implanted in addition to the Absorb BVS, in cases where Absorb BVS could not be implanted due to the unavailability of the required device size. Finally, a longer follow-up is mandatory to further evaluate the performance of the Absorb BVS in this specific group of patients.

## Conclusion

The results of this multinational dedicated prospective study show that PCI with Absorb BVS for treatment of non-extensive CAD in DM patients, performed by experienced operators, is associated with acceptable safety and efficacy outcomes at 1 year when historically comparable to modern DES. If maintained for longer follow-up periods, covering the resorption phase of the device, our study results would further reinforce the potentially advantageous impact of bioresorbable scaffolds for CAD treatment in fast progressing atherosclerosis populations like DM patients, where PCI even with modern DES remains challenging. From this perspective, it could pave the way for further research with new-generation and more performant bioresorbable scaffolds.

## Caption Electronic Supplementary Material

A detailed explanation of the adverse event definitions is formulated in the Supplementary Table.
