# Supplementary material for: The 1‑year safety and efficacy outcomes of Absorb bioresorbable vascular scaffolds for coronary artery disease treatment in diabetes mellitus patients: the ABSORB DM Benelux study
Source: Neth Heart J. 2019 Jun 13;27(11):541–9. doi: 10.1007/s12471-019-1293-7 (PMC6823340; doi:10.1007/s12471-019-1293-7)
Supplement: Supplementary file 1 — A detailed explanation of the adverse event definitions is formulated in the Supplementary Table. [file 12471_2019_1293_MOESM1_ESM.docx]

**Supplementary table**

All-cause death was determined as death to any cause. CD was defined as any death due to immediate cardiac cause like MI, arrhythmia or congestive heart failure. Unwitnessed death, death due to unknown cause, death secondary to cerebrovascular accident or death related to PCI or CABG, were all classified as CD. MI deﬁnitions were defined according to the universal deﬁnition of MI [1]. TVR was defined as any repeat PCI or CABG of the target vessel of any segment of the target vessel. The latter was considered as the entire major coronary vessel proximal and distal from target lesion including side branches. TLR was defined as any repeat PCI or CABG of the target vessel performed for restenosis or other complication of the target lesion. The target lesion was defined as the treated segment from 5 mm proximal and 5 mm distal to the scaffold.

A revascularisation was reckoned as ischaemia-driven if: (i) angiography showed a diameter stenosis ≥50% on quantitative coronary angiography and if a single of the following criteria was met: a positive history of recurrent angina pectoris presumably related to the target vessel or objective signs of ischaemia at rest (electrocardiogram changes) or during exercise test (or equivalent) presumably related to the target vessel; (ii) abnormal results of any invasive functional diagnostic test; (iii) presence of a ruptured coronary atherosclerotic lesion with or without adjacent thrombus on intracoronary imaging evaluation in the presence of clinical symptoms which were to be related to an acute coronary syndrome. Further general definitions were defined as described in the ACC/AHA Clinical Data Standards [2]. ScT was deﬁned according to the Academic Research Consortium [3].

1. Thygesen K, Alpert JS, Jaffe AS, et al. Third universal deﬁnition of myocardial infarction. J Am Coll Cardiol. 2012;60:1581-98.

2. Hicks KA, Tcheng JE, Bozkurt B, et al. 2014 ACC/AHA key data elements and definitions for cardiovascular endpoint events in clinical trials: a report of the American College of Cardiology/American Heart Association task force on clinical data standards (writing committee to develop cardiovascular endpoints data standards). J Nucl Cardiol. 2015;22:1041-144.

3. Cutlip DE, Windecker S, Mehran R, et al. Clinical end points in coronary stent trials: a case for standardized deﬁnitions. Circulation. 2007;115:2344-51.
